# Effect of Ketamine on Limbic GABA and Glutamate: A Human *In Vivo* Multivoxel Magnetic Resonance Spectroscopy Study

**DOI:** 10.3389/fpsyt.2020.549903

**Published:** 2020-09-08

**Authors:** Leo R. Silberbauer, Benjamin Spurny, Patricia Handschuh, Manfred Klöbl, Petr Bednarik, Birgit Reiter, Vera Ritter, Patricia Trost, Melisande E. Konadu, Marita Windpassinger, Thomas Stimpfl, Wolfgang Bogner, Rupert Lanzenberger, Marie Spies

**Affiliations:** ^1^ Department of Psychiatry and Psychotherapy, Medical University of Vienna, Vienna, Austria; ^2^ Department of Biomedical Imaging and Image-guided Therapy, High Field MR Centre, Medical University of Vienna, Vienna, Austria; ^3^ Department of Laboratory Medicine, Medical University of Vienna, Vienna, Austria; ^4^ Department of Anesthesia, Intensive Care Medicine and Pain Medicine, Medical University of Vienna, Vienna, Austria

**Keywords:** ketamine, GABA, glutamate, magnetic resonance spectroscopy, depression, ketamine metabolites, limbic system

## Abstract

**Introduction:**

Converging evidence suggests that ketamine elicits antidepressant effects *via* enhanced neuroplasticity precipitated by a surge of glutamate and modulation of GABA. Magnetic resonance spectroscopic imaging (MRSI) illustrates changes to cerebral glutamate and GABA immediately following ketamine administration during dissociation. However, few studies assess subacute changes in the first hours following application, when ketamine’s antidepressant effects emerge. Moreover, ketamine metabolites implicated in its antidepressant effects develop during this timeframe. Thus, this study aimed to investigate subacute changes in cerebral Glx (glutamate + glutamine), GABA and their ratio in seven brain regions central to depressive pathophysiology and treatment.

**Methods:**

Twenty-five healthy subjects underwent two multivoxel MRS scans using a spiral encoded, MEGA-edited LASER-localized 3D-MRSI sequence, at baseline and 2 h following intravenous administration of racemic ketamine (0.8 mg/kg bodyweight over 50 min). Ketamine, norketamine and dehydronorketamine plasma levels were determined at routine intervals during and after infusion. Automated region-of-interest (ROI)–based quantification of mean metabolite concentration was used to assess changes in GABA+/total creatine (tCr), Glx/tCr, and GABA+/Glx ratios in the thalamus, hippocampus, insula, putamen, rostral anterior cingulate cortex (ACC), caudal ACC, and posterior cingulate cortex. Effects of ketamine on neurotransmitter levels and association with ketamine- and metabolite plasma levels were tested with repeated measures analyses of variance (rmANOVA) and correlation analyses, respectively.

**Results:**

For GABA+/tCr rmANOVA revealed a measurement by region interaction effect (p_uncorr_ < 0.001) and *post hoc* pairwise comparisons showed a reduction in hippocampal GABA+/tCr after ketamine (p_corr_ = 0.02). For Glx/tCr and GABA+/Glx neither main effects of measurement nor measurement by region interactions were observed (all p_uncorr_ > 0.05). Furthermore, no statistically significant associations between changes in any of the neurotransmitter ratios and plasma levels of ketamine, norketamine, or dehydronorketamine were observed (p_corr_ > 0.05).

**Conclusion:**

This study provides evidence for decreased hippocampal GABA+/tCr ratio 2 h following ketamine administration. As MRS methodology measures total levels of intra- and extracellular GABA, results might indicate drug induced alterations in GABA turnover. Our study in healthy humans suggests that changes in GABA levels, particularly in the hippocampus, should be further assessed for their relevance to ketamine´s antidepressant effects.

## Introduction

As a rapid acting and robust glutamatergic agent, ketamine occupies a unique position in antidepressant therapy. Numerous randomized controlled trials substantiate its efficacy in uni- and bipolar depression (1–3). The recent approval of an intranasal formulation by the U.S. Food and Drug Administration (FDA) and the European Medicines Agency (EMA) is likely to promote the use of ketamine in clinical care.

The glutamate model of depression implicates alterations to glutamate- and γ-aminobutyric acid (GABA)-related synaptic function (4); stress-related dysfunctional glutamate cycling is thought to result in excitotoxicity and neuronal atrophy. N-methyl-D-aspartate (NMDA)-receptor-mediated inhibition of GABA interneurons, resulting in reduced inhibitory control over prefrontal glutamate neurons and subsequent glutamate burst, are thought to facilitate ketamine’s antidepressant effects (5). Accompanying α-amino-3-hydroxy-5-methyl-4-isoxazolepropionic acid (AMPA)-receptor agonism activates second messenger pathways implicated in neuroplasticity (6). Thus, assessing brain glutamate and GABA levels provides insight into ketamine’s antidepressant mechanisms of action.

Proton magnetic resonance spectroscopy (^1^H-MRS) enables measurement of total tissue concentrations of glutamate (Glu), glutamine (Gln), pooled Glu and Gln (Glx) and GABA, among other metabolites in the living human brain. Gln is both glutamate precursor and metabolite; after Glu release, glia convert Glu to Gln, which is cycled back to the neuron (7). Consequently, Glx might be understood as an index of total glutamatergic potential (8).

MRS performed during or immediately after ketamine infusion shows increases in prefrontal cortex (PFC) glutamate (9–13), which is likely responsible for dissociative symptoms (14). Studies on the acute (up to 30 min postinfusion) influence of ketamine on GABA levels are currently limited. Two studies demonstrated increased PFC GABA levels (11, 15), though no change was observed in the thalamus (10).

Antidepressant effects emerge 2 h after infusion when acute effects, such as dissociation, have worn off and peak after 24 h (16, 17). Estimation of Glx and GABA within a timeframe when antidepressant effects become evident is essential to understanding their relevance in ketamine’s antidepressant properties. Previous studies on subacute changes show increase in pregenual anterior cingulate cortex (pgACC) Gln/Glu ratio after 24 h, which was not yet detected 1 h postinfusion (18, 19). Two other studies did not observe changes in Glu, Gln, Gln/Glu (20), nor GABA at various time points up to 48 h postinfusion (21). Interestingly, ketamine metabolites norketamine (norket) and dehydronorketamine (dhnk), to which antidepressant effects have been attributed in preclinical trials (22, 23), begin to develop during this time period (24). However, little is known about their glutamatergic and GABAergic effects.

Thus, research on ketamine’s glutamate and GABA effects during this postdissociative timeframe, which is characterized by clinical antidepressant improvement, are currently limited. Furthermore, existing studies are restricted to single-voxel MRS approaches that do not allow for concomitant assessment of glutamate and GABA in multiple brain regions. However, we recently illustrated feasibility of a novel multivoxel 3D-magentic resonance spectroscopy imaging (MRSI) sequence with MEGA-LASER editing for parallel assessment of multiple regions of interest (25–27). In addition to propagating a topographically more extensive assessment of ketamine’s effects, this multivoxel MRSI sequence allows for concomitant assessment of Glx and GABA+ (a combination of GABA and macromolecules), and thus also of their relation. Dysfunctionality of this relation has been discussed in the context of depressive pathophysiology (5, 8).

Here, we leverage 3D multivoxel MRSI technology to investigate ketamine’s effects on Glx, GABA+, and their ratio in limbic brain regions implicated in depressive pathophysiology at a time when antidepressant effects of ketamine emerge. Regionally specific assessment during this time provides insight into the chronology of ketamine’s neurotransmitter effects and their relevance for ketamine’s antidepressant properties.

## Methods

### Subjects

Twenty-five healthy male subjects (mean age ± SD = 26.76 ± 4.71) were included in these analyses. Volunteers were recruited *via* postings on dedicated message boards at the Medical University of Vienna. Participants were free from internal, neurological or psychiatric disorders assessed *via* a thorough medical history, physical examination, electrocardiogram and routine laboratory parameters. The Structured Clinical Interview for DSM-IV Axis-I Disorders (SCID I) was administered by a psychiatrist in order to exclude any previous or current psychiatric diagnoses. Individuals had no history of substance use disorder and urine drug tests were performed at screening and each magnetic resonance imaging (MRI) session to exclude current drug use. Subjects were excluded at screening if they had any MRI contraindications. All participants provided written informed consent and received financial reimbursement for their participation. This study was approved by the Ethics Committee of the Medical University of Vienna and carried out according to the Declaration of Helsinki.

### Study Design and Ketamine Administration

Subjects underwent two MRI measurements. While the first measurement (MRI1) was performed without pharmacological challenge and served as a baseline scan, participants received 0.8 mg/kg bodyweight racemic ketamine (Ketamine hydrochloride, 50 mg/ml ampoules, Hameln Pharma Plus GmbH) intravenously over the course of 50 min starting 120 min prior to the second scan (MRI2) (see **Figure S1**). The dose of 0.8 mg/kg was chosen as it is within the subanesthetic, antidepressant dose range (1, 2, 28). Vital parameters were monitored regularly and a clinician was present at all times.

### Magnetic Resonance Imaging

MRI measurements were performed using a 64-channel head coil on a 3 Tesla MR Scanner (MAGNETOM Prisma, Siemens Medical, Erlangen, Germany) installed at the High-field MR Center, Department of Biomedical Imaging and Image-guided Therapy, Medical University of Vienna. Structural T1‐weighted images were acquired during each measurement using a standard magnetization‐prepared rapid gradient‐echo (MPRAGE) sequence (TE = 1800 ms, TR = 2.37 ms, 208 slices, 288 × 288 matrix size, slice thickness 0.85 mm, voxel size 1.15 × 1.15 × 0.85 mm) for accurate placement of the volume of interest (VOI) and mask extraction for automated region of interest (ROI)–based analysis.

**Figure 1:**
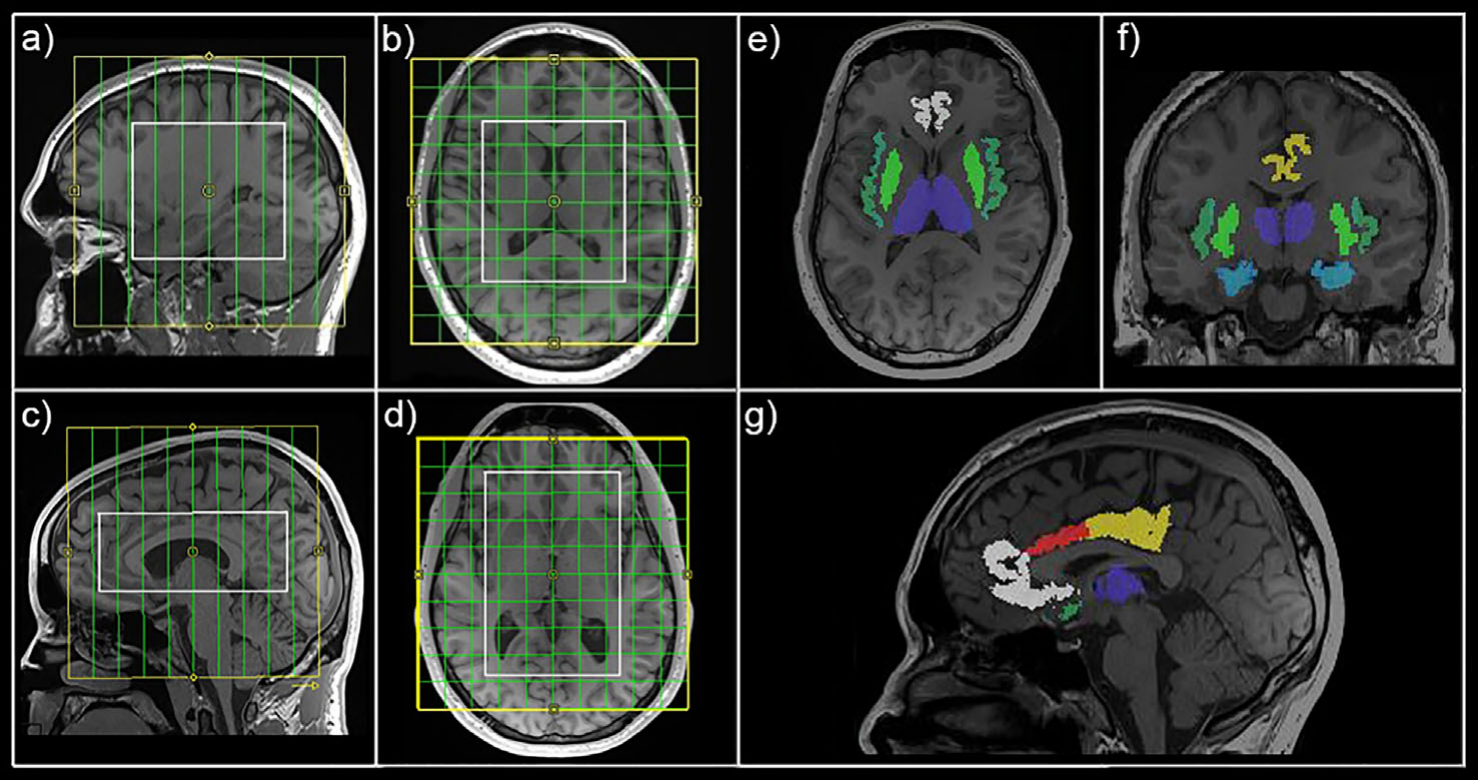
Magnetic resonance spectroscopic imaging (MRSI) position and mask extraction. Field of view (yellow) and volume of interest (white) are shown for MRSI1 **(A**, **B)** and MRSI2 **(C**, **D)** in sagittal and horizontal view, respectively. Exemplary automated mask extraction with FreeSurfer is shown in horizontal **(E)**, coronal **(F)**, and sagittal **(G)** view showing masks for the thalamus (purple), hippocampus (light blue), insula (dark green), putamen (light green), rostral anterior cingulate cortex (white), caudal anterior cingulate cortex (red), and posterior cingulate cortex (yellow).

For spectroscopic measurements, a constant‐density, spiral‐encoded, 3D‐MRSI sequence with MEGA‐LASER editing, as described in (25), was used. Real‐time correction for rigid-body motion bias (i.e., translations and rotations) and correction of center frequency changes was applied (25, 29). Two MRSI measurements (MRSI1 and MRSI2) were performed consecutively to cover all ROIs and avoid inclusion of lipid-rich regions (see **Figure 1**). All MRS slices were placed parallel to the anterior commissure–posterior commissure line. Position of the VOIs of the second scanning session were determined based on VOI placement of the baseline measurement. VOI1 was centered to the medial part of the corpus callosum to cover the hippocampus and insula bilaterally, with a VOI = 80 (l-r) × 90 (a-p) × 80 (s-i) mm^3^ and a field of view (FOV) = 160 × 160 × 160 mm^3^, see **Figure 1**. The acquired matrix size of 10 × 10 × 10 (i.e., ~4 cm^3^ nominal voxel size) was interpolated to a 16 × 16 × 16 matrix (i.e., ~1 cm^3^ nominal voxel size) during spectral processing steps. VOI2 was centered to cover the cingulate cortex with VOI = 80 (l-r) × 120 (a-p) × 50 (s-i) mm^3^ and field of view (FOV) = 160 × 160 × 160 mm^3^, see **Figure 1**. Siemens advanced shimming procedure with manual adjustments was used. During the EDIT‐ON acquisition, MEGA‐editing pulses (60 Hz Gaussian pulses of 14.8 ms duration) were set to 1.9 ppm, editing the coupled 4CH_2_ triplet of GABA resonating at 3.02 ppm (30, 31). VOI selection *via* LASER and low‐power and wide‐bandwidth GOIA pulses enabled MEGA editing with an echo time of 68 ms (25). For real‐time correction, volumetric, dual‐contrast, echo planar imaging-based navigators that update center frequency and head‐position changes for each pair of EDIT‐ON/OFF acquisitions were used (i.e., with a repetition time of 1.6 s, updated every 3.2 s). For 3D‐MRSI, 32 acquisition-weighted averages and two‐step phase cycling were employed, resulting in a total scan time of 15:09 min per MRSI scan.

### Magnetic Resonance Spectroscopy Data Analyses

All spectra within the VOI were processed automatically with an in-house software tool using MATLAB (R2013a, MathWorks, Natick, MA, USA), Bash (4.2.25, Free Software Foundation, Boston, MA, USA), MINC (2.0, MINC Tools, McConnell Brain Imaging Center, Montreal, QC, Canada) and LCModel software (6.3–1, S. Provencher, LCModel, Oakville, ON, Canada). Two different simulated basis sets were created using the GAMMA library, one for the non-edited spectra [containing 21 brain metabolites, including total creatine (tCr)] and one for the difference spectrum (containing GABA+ and Glx among others) (32, 33) (see **Figure S2** for exemplary spectra). Cramér-Rao lower bounds (CRLB) thresholds were set at 30%. GABA+ and Glx ratios were calculated relatively to tCr (GABA+/tCr and Glx/tCr). Automated ROI-based analyses were performed as previously described (26). FreeSurfer (6.0, https://surfer.nmr.mgh.harvard.edu/) (34, 35) was used for automated segmentation of structural T1-weighted images (see **Figure 1**). In-house MATLAB code was used to extract the ROIs (thalamus, hippocampus, insula, putamen, rostral anterior cingulate cortex (rACC), caudal anterior cingulate cortex (cACC), posterior cingulate cortex (PCC)) from each measurement. ROIs contained at least 2 voxels from the original 16 × 16 × 16 grid (see **Table S1** for volumetric properties of derived masks). GABA+ and Glx (derived from the non-edited spectra) and tCr maps were interpolated to the resolution of structural images, overlaid with masks and mean GABA+/tCr, Glx/tCr and GABA+/Glx ratios calculated for each ROI. Metabolic data for thalamus, hippocampus, insula and putamen were derived from MRSI1 and rACC, cACC, and PCC from MRSI2. ROIs with <90% valid interpolated voxels were excluded from analyses for the particular subject. ROIs were chosen based on their relevance to depressive pathophysiology, especially in the context of the glutamatergic depression model (5). rACC and cACC were investigated separately based on previous reports of ACC subregion-specific effects (18, 36).

### Ketamine and Metabolite Plasma Levels

Venous blood samples were drawn at baseline and 20, 40, 50, 55, 60, 70, 80 min after ketamine administration, as well as immediately prior to and after MRI2. After centrifugation and separation of plasma, samples were frozen at −80°C until analysis. Determination of ketamine, norket and dhnk plasma levels was accomplished using gas chromatography-mass spectrometry (GC-MS/MS) at the Clinical Department of Laboratory Medicine, Medical University of Vienna, Austria. The applied method was validated according to the European Medicines Agency (EMA) guideline on bioanalytical method validation (37).

### Statistical Analyses

SPSS Statistics (v26.0, 2010, SPSS, Inc., an IBM Company, Chicago, United States of America) and MATLAB (R2011a, MathWorks, Natick, MA, USA) were used for statistical analyses. Repeated measures analyses of variance (rmANOVA) with metabolite (GABA+/tCr, Glx/tCr or GABA+/Glx) as dependent variable and measurement (MRI1, MRI2) and ROI (thalamus, hippocampus, insula, putamen, rACC, cACC, PCC) as within-subject factors were performed to probe for effects of ketamine on metabolite ratios. Interaction (measurement by region) and main effects (measurement, region) were tested.

Missing ratio values that failed to pass quality criteria (CRLB thresholds) were estimated using multiple imputation using ten repetitions and mean imputed values were used in subsequent analyses. See **Table 1** for extent of imputed values per ROI and metabolite ratio. rACC was excluded from GABA+/tCr and GABA+/Glx models because missing values exceeded 40%. Each metabolite ratio was estimated in an individual model. In case of significant main effect of measurement or interaction effects, *post hoc* pair-wise comparisons (t-test) were performed to isolate ROI-specific measurement effects. For Glx/tCr ratio, residuals were not consistently normally distributed (based on visual inspection of histograms). Logarithmic transformation of Glx/tCr ratios was performed and rmANOVA was repeated, after which residuals showed normal distribution. Bonferroni procedure was used to correct for multiple comparisons. Associations between changes in neurotransmitter ratio from MRI1 to MRI2 (dependent variable) and ketamine, norket, and dhnk plasma levels (independent variables) were probed with spearman correlation analyses, as dependent variables did not show normal distribution (based on visual inspection of histograms). Plasma levels were interpolated to the time point of the start of each MRSI measurement for each subject using linear interpolation in MATLAB. Correlation analyses were performed for each ROI, neurotransmitter ratio and plasma level time-course. Again, Bonferroni procedure was utilized to correct for multiple comparisons. Ketamine, norket, and dhnk samples were available in 20 subjects. Raw MRSI data, without imputed values (see **Table 1**) was used to calculate MRI1 to MRI2 difference. As a result, sample sizes of correlation analysis were limited and we only report on ROIs with ≥ 10 available data points. This was not the case for GABA+/tCr and GABA+/Glx ratios in the hippocampus, insula and rACC.

**Table 1:** Number of imputed values, split by region of interest (ROI) and measurement.

	GABA+/tCr	CRLB (%)	Glx/tCr	CRLB (%)	GABA+/Glx
Thalamus MRI1	1 (4%)	12.98	0 (0%)	8.60	1 (4%)
Thalamus MRI2	4 (16%)	12.91	3 (12%)	9.31	3 (12%)
Hippocampus MRI1	8 (32%)	17.18	1 (4%)	11.22	8 (32%)
Hippocampus MRI2	9 (36%)	17.66	3 (12%)	13.16	9 (36%)
Insula MRI1	5 (20%)	17.20	0 (0%)	10.69	5 (20%)
Insula MRI2	9 (36%)	16.38	3 (12%)	11.41	9 (36%)
Putamen MRI1	4 (16%)	13.22	0 (0%)	9.28	4 (16%)
Putamen MRI2	9 (36%)	14.38	3 (12%)	10.28	9 (36%)
rACC MRI1	17 (68%)	> 30	7 (28%)	13.44	17 (68%)
rACC MRI2	15 (60%)	> 30	4 (16%)	13.32	15 (60%)
cACC MRI1	9 (36%)	16.25	6 (24%)	9.85	9 (36%)
cACC MRI2	9 (36%)	15.65	3 (12%)	9.11	9 (36%)
PCC MRI1	9 (36%)	15.84	6 (24%)	9.31	9 (36%)
PCC MRI2	9 (36%)	15.94	3 (12%)	8.54	9 (36%)

Number of values that did not meet quality criteria based on CRLB thresholds and were imputed for analysis. More than 40% of rACC GABA+ values failed quality criteria and the ROI was thus excluded from statistical analyses. GABA+, GABA and macromolecules; Glx, glutamate + glutamine; tCr, total creatine; rACC, rostral anterior cingulate cortex; cACC, caudal anterior cingulate cortex; PCC, posterior cingulate cortex; CRLB, Cramér-Rao lower bounds.

## Results

**Figure 2:**
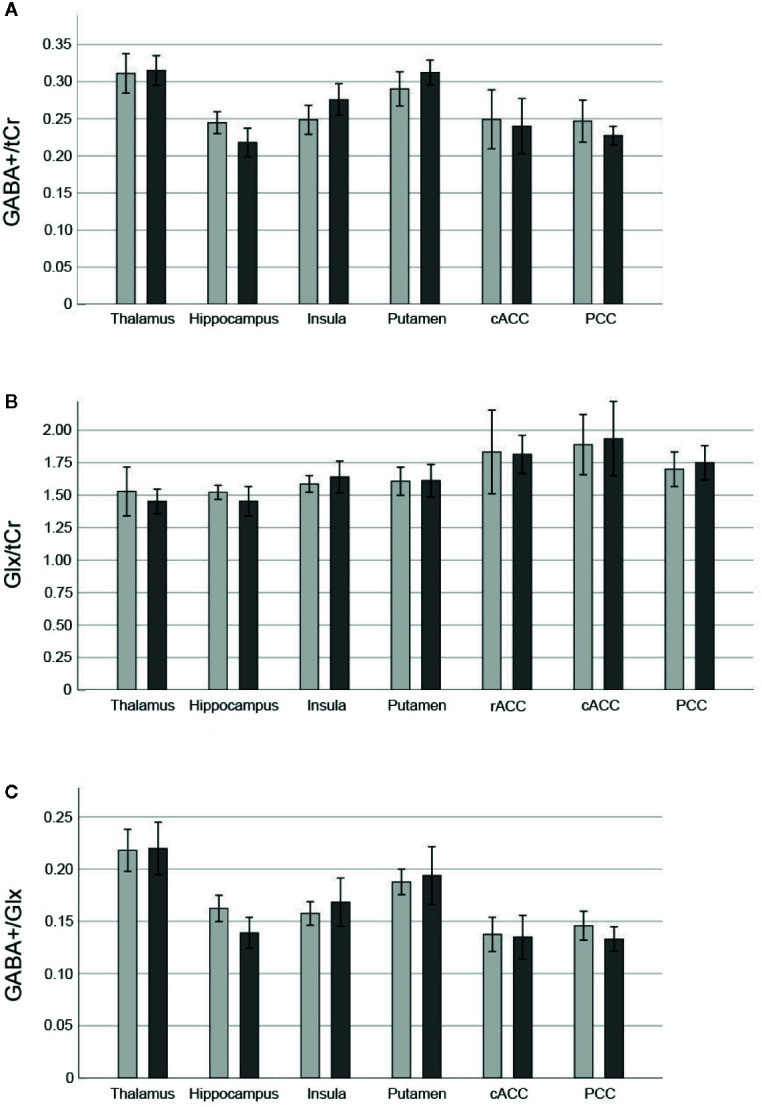
Neurotransmitter ratios before and after ketamine. Bars denote mean neurotransmitter ratios [GABA+/tCr **(A)**, Glx/tCr **(B)**, GABA+/Glx **(C)**] before (light grey) and 2 h after infusion of 0.8 mg/kg bodyweight racemic ketamine (dark grey), brackets denote two standard errors. GABA+ values in the rACC did not meet quality criteria based on CRLB in more than 40% of cases and thus were excluded from statistical analyses. GABA+, GABA and macromolecules; Glx, glutamate + glutamine; tCr, total creatine; rACC, rostral anterior cingulate cortex; cACC, caudal anterior cingulate cortex; PCC, posterior cingulate cortex; CRLB, Cramér-Rao lower bounds.

**Figure 3:**
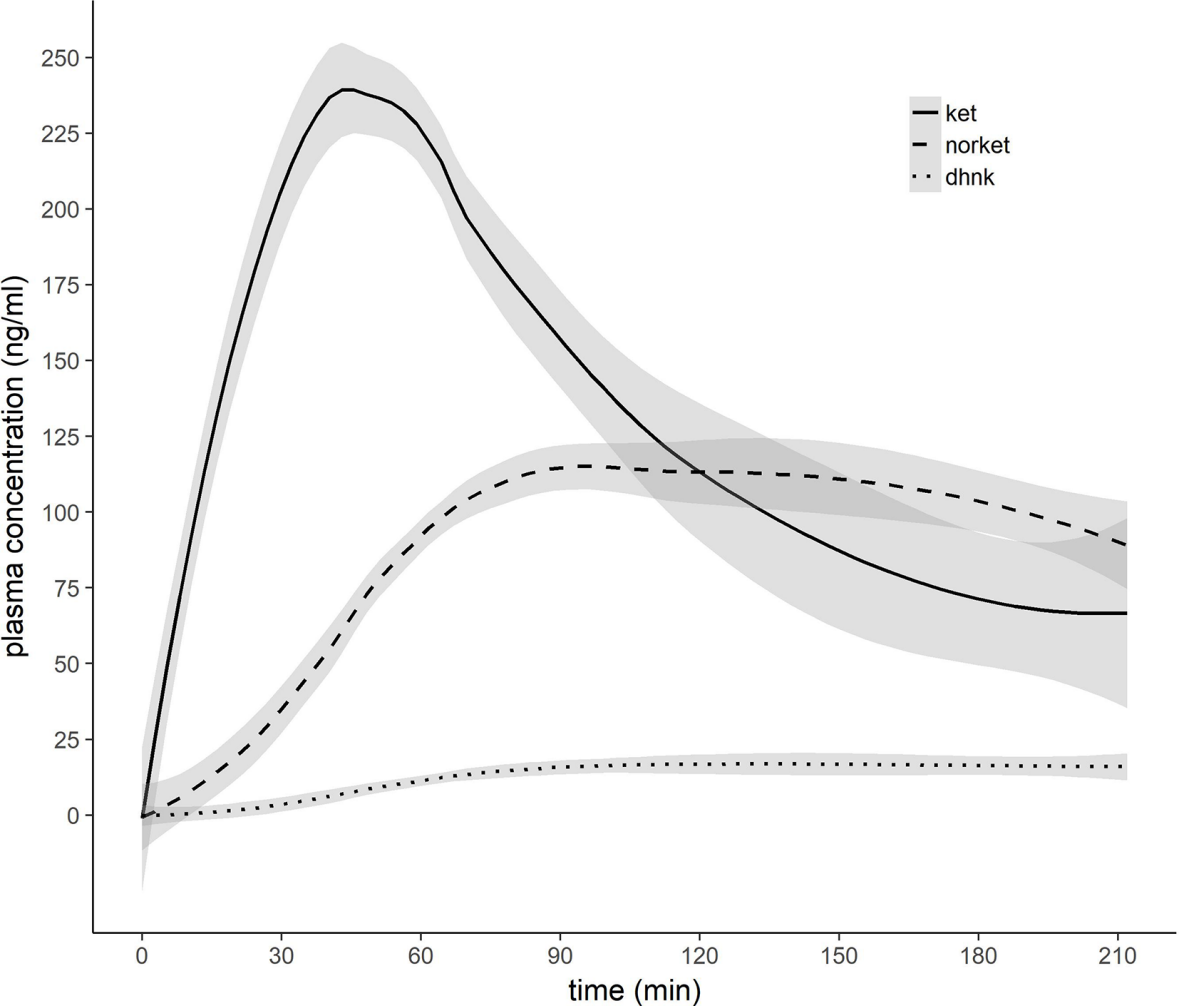
Pharmacokinetic profile of ketamine, norketamine, and dehydronorketamine. 0.8 mg/kg bodyweight racemic ketamine were infused over 50 min. X-axis indicates minutes after initiation of ketamine infusion. Grey ribbons indicate standard error of the mean. Ket, ketamine; norket, norketamine; dhnk, dehydronorketamine.

Mean ± SD time between MRI1 and MRI2 was 67.84 ± 72.43 days. Mean ± SD time between start of ketamine administration and MRSI data acquisition was 137.44 ± 3.43 min. See **Table 2** for mean ± SD GABA+/tCr, Glx/tCr, and GABA+/Glx for each ROI and measurement. Neurotransmitter ratios before and after ketamine application are displayed in **Figure 2**. See **Table S2** and **Figure 3** for average ketamine, norket, and dhnk plasma levels.

**Table 2:** Mean GABA+/tCr, Glx/tCr and GABA+/Glx ratios split by region of interest (ROI) and measurement.

		Thalamus	Hippocampus	Insula	Putamen	rACC	cACC	PCC
	MRI1							
GABA+/tCr	mean ± SD	0.31 ± 0.07	0.24 ± 0.03	0.25 ± 0.04	0.29 ± 0.05		0.25 ± 0.08	0.25 ± 0.06
	MRI2							
	mean ± SD	0.32 ± 0.05	0.22 ± 0.04	0.28 ± 0.04	0.31 ± 0.03		0.24 ± 0.07	0.23 ± 0.03
	difference (%)	1.29	−10.85	10.97	7.64		−3.68	−7.91
	MRI1							
Glx/tCr	mean± SD	1.53 ± 0.47	1.52 ± 0.13	1.59 ± 0.16	1.61 ± 0.27	1.83 ± 0.68	1.89 ± 0.51	1.70 ± 0.29
	MRI2							
	mean± SD	1.45 ± 0.22	1.45 ± 0.26	1.64 ± 0.29	1.61 ± 0.29	1.81 ± 0.34	1.94 ± 0.67	1.75 ± 0.31
	difference (%)	−5.05	−4.52	3.41	0.28	−1.00	2.46	2.93
	MRI1							
GABA+/Glx	mean ± SD	0.22 ± 0.05	0.16 ± 0.03	0.16 ± 0.02	0.19 ± 0.03		0.14 ± 0.03	0.15 ± 0.03
	MRI2							
	mean ± SD	0.22 ± 0.06	0.14 ± 0.03	0.17 ± 0.05	0.19 ± 0.06		0.13 ± 0.04	0.13 ± 0.02
	difference (%)	0.85	−14.41	6.84	3.30		−1.87	−8.81

Raw data without imputed values is used; rACC GABA+/tCr and GABA+/Glx values did not meet quality criteria based on CRLB and were thus excluded from statistical analyses. GABA+, GABA and macromolecules; Glx, glutamate + glutamine; tCr, total creatine; rACC, rostral anterior cingulate cortex; cACC, caudal anterior cingulate cortex; PCC, posterior cingulate cortex; CRLB, Cramér-Rao lower bounds.

RmANOVA of GABA+/tCr showed a significant measurement by region interaction effect (F_4.04,96.86_ = 6.17, p_uncorr_ < 0.001), significant effect of region (F_3.43,82.36_ = 34.27, p_uncorr_ < 0.001), but no significant effect of measurement (F_1,24_ = 0.19, p_uncorr_ = 0.67). *Post hoc* pair-wise comparison revealed significantly higher GABA+/tCr at MRI1 than MRI2 in the hippocampus (p_corr_ = 0.02), effects in insula (p_uncorr_ = 0.03), putamen (p_uncorr_ = 0.02), and PCC (p_uncorr_ = 0.03), did not survive correction for multiple comparisons. For Glx/tCr and GABA+/Glx neither measurement by region interaction effects (Glx/tCr: F_3.46,82.92_ = 0.85, p_uncorr_ = 0.48; GABA+/Glx: F_2.87,68.76_ = 2.34, p_uncorr_ = 0.08), nor main effects of measurement (Glx/tCr: F_1,24_ = 0.03, p_uncorr_ = 0.87; GABA+/Glx: F_1,24_ = 1.05, p_uncorr_ = 0.32) were detected, though main effects of region were statistically significant (Glx/tCr: F_3.37,80.83 =_ 11.61, p_uncorr_ < 0.001; GABA+/Glx: F_2.45,58.91_ = 44.79, p_uncorr_ < 0.001).

Based on Mauchly’s test sphericity could not be assumed; Greenhouse-Geisser corrected results are given.

Before correction for multiple comparisons, correlation analyses revealed significant positive associations between GABA+/tCr difference MRI1 to MRI2 and ketamine plasma level (ρ = 0.66, p_uncorr_ = 0.04) in the cACC, and with norket plasma level in the putamen (ρ = 0.84, p_uncorr_ = 0.002), as well as difference in Glx/tCr from MRI1 to MRI2 and dhnk plasma level in the hippocampus (ρ = 0.53, p_uncorr_ = 0.03). However, none of these effects survived correction for multiple comparisons.

## Discussion

This multivoxel MRSI study assessed *in vivo* Glx/tCr, GABA+/tCr, and GABA+/Glx 2 h following intravenous ketamine infusion in brain regions relevant to the glutamatergic theories of depression and antidepressant efficacy. We detected a significant reduction of GABA+/tCr ratio in the hippocampus. No statistically significant changes in Glx/tCr and GABA+/Glx ratios were observed. To the best of our knowledge, this is the first multivoxel MRS study to demonstrate subacute (2 h) effects of ketamine administration on limbic GABA levels in humans *in vivo*.

Timing MRS 2 h after ketamine administration assesses potential impact when dissociative effects wear off and ketamine´s antidepressant properties unfold (16, 17). Thus, in theory, changes during this time period might be considered a temporally more related correlate of the substance´s antidepressant effects. Despite this argument, previous human ketamine MRS studies on GABA levels were mostly limited to reports on immediate drug effects. While Stone et al. (10) did not observe acute changes in GABA following ketamine administration, two studies demonstrated increased GABA levels in the PFC during (11) and immediately after ketamine infusion (15). So far, the only study investigating subacute effects of ketamine on GABA concentration was performed by Valentine et al. (21). In this study, neither a change in occipital GABA, Glu nor Gln was observed 3 or 48 h after ketamine infusion in MDD. Our study differs from Valentine et al. (21) in that we assessed the hippocampus, among other regions, rather than the occipital cortex. These regional differences bring to light the importance of a regionally extensive investigation. Using multivoxel MRSI, seven ROIs were selected based on their relevance to depressive pathophysiology. Though assessment of several ROIs as we did also requires a-priori selection, bias is likely nevertheless lower than that resulting from single-voxel investigations which limit results and their interpretation to a single region. In addition, we assessed healthy controls rather than patients with MDD. Though this limits the extent to which changes we observe can be interpreted as direct facilitators of antidepressant efficacy, our investigation highlights changes that should be assessed in future studies in MDD. In addition, our focus on ROIs that are central to depressive pathophysiology increases potential for detection of clinically relevant changes that can then be validated in future patient studies.

Our current data may at first appear inconsistent with previous reports of reduced GABA levels in depression (38) and normalization following antidepressant treatment such as with selective serotonin reuptake inhibitors and electroconvulsive therapy (39, 40). Given the sparse human data on subacute effects of ketamine on GABA, preclinical data may provide guidance on this point. Increases in GABA levels were reported 30 min but not 24 h after ketamine administration in prefrontal regions in rats (41). Perrine et al. (42) reported decreased GABA levels 24 h after ketamine administration in the ACC. Moreover, reduced GABA in parvalbumin (PV) interneurons of the PFC was demonstrated 2 h after ketamine administration by Zhou et al. (43). Thus, various preclinical studies suggest GABA reduction within the subacute timeframe (2–24 h). In accordance with our hippocampus findings, Wang et al. (44) reported immediate decreases in GABA levels in hippocampal PV interneurons in rats following ketamine. Decreases in GABA levels as reported by Wang et al. (44) and (43) were associated with downregulation of GAD67, a protein implicated in the glutamate/GABA-glutamine cycle, suggesting alterations in GABA turnover (45). Thus, though in contrast with some human *in vivo* reports, our results are in line with various preclinical studies suggesting subacute GABA reduction after ketamine.

We did not detect statistically significant effects on Glx/tCr. Preclinical studies report a rapid glutamate surge immediately following ketamine administration (5). Human MRS demonstrates concomitant changes in Glu, Gln and Glx levels (9–13). However, similar to GABA, previous studies on subacute effects of ketamine on Glu and Gln are also limited. Evans et al. (20) did not detect changes in Glu, Gln or Gln/Glu in the pgACC 24 h after ketamine administration in MDD. In contrast, Li et al. report increased ACC Gln/Glu 24 h after infusion in healthy individuals. This effect was not yet present at 1 h and was restricted to the pgACC (18, 19). Li et al. interpret this regional specificity to be reflective of AMPA distribution, more specifically AMPA/NMDA ratio, which is higher in the pgACC compared to anterior midcingulate cortex (aMCC) (36, 46). We did not detect regional differences within the ACC after 2 h, though our ROIs (rACC, cACC) do not entirely overlap with pgACC and aMCC as delineated by (18, 19).

MRS ketamine literature exhibits heterogeneous outcome parameters; we assessed GABA+/tCr, Glx/tCr, and GABA+/Glx. With the exception of Gln/Glu, which is considered indicative of alterations in turnover and seen as an index of release (47), MRS directly assesses changes in total neurotransmitter levels; alterations to relative intra/extracellular concentrations, as would be postulated in release or cell-cell (neuron–glial) cycling, are not primarily detected. Any effects on Glx/tCr or GABA+/tCr are reflective of change in total concentration, for example *via* alterations in turnover. The decreased GABA+/tCr ratio observed in this study might be interpreted within this context. Synthesis and degradation of GABA and glutamate within the glutamate/GABA-glutamine cycle are shown to be closely tied to the tricarboxylic acid (TCA) cycle, which is central to oxidative energy metabolism (7). Changes in GABA and glutamate concentrations likely occur dynamically and in an activity-dependent manner on a short timescale of min (11, 48). Thus, it is plausible that ketamine might induce changes in total concentration of Glx and GABA detectable with MRS within our measurement timeframe. Along this line, rodent studies suggest a modest acute increase in *de novo* glutamate (~18%) and GABA (~10%) synthesis following ketamine administration in the medial PFC (mPFC). No changes, however, were observed in the hippocampus (49). Though our human *in vivo* MRS study detected decreases in hippocampal GABA+/tCr ratio, the aforementioned studies highlight that changes to GABA or glutamate turnover are feasible within our measurement timeframe. The only previous MRS studies showing subacute glutamatergic alterations after ketamine infusion assess Gln/Glu (18, 19). However, changes in Gln/Glu could coincide with constant Glx, in which case they would not be observed in our study.

We investigated possible changes in GABA+/Glx ratio based on previous evidence of imbalanced excitatory and inhibitory neurotransmission in depressive pathophysiology and in relation to antidepressant efficacy (50). Previous studies indicate that rapid acting antidepressants might restore deficits in this balance by enhancing GABAergic neurotransmission (51). While we find downregulation of hippocampal GABA levels within the subacute period, we did not detect a statistically significant effect of ketamine on GABA+/Glx ratios in any ROI. Thus, our results do not provide evidence that ketamine infusion affects balance of GABA and glutamate levels 2 h after administration. Given ketamine’s synchronized glutamatergic and GABAergic effects (5), studies on GABA+/Glx at other time points should be assessed in future studies.

We specifically probed alterations to GABA+/tCr, Glx/tCr, and GABA+/Glx because we postulated that assessment of changes during the period of clinical efficacy may provide insight into antidepressant mechanisms of action. While decreased hippocampal GABA/tCr sheds light on the importance of the GABAergic system in the antidepressant efficacy of ketamine, other therapeutic mechanisms of action have previously been shown to be relevant. Most often discussed among these processes is neuroplasticity activated by modulation of the glutamate and GABA systems. Ketamine administration increases spine formation by activation of the mTOR pathway in rodents (52), a process that is considered to reverse neuronal atrophy associated with depressive pathophysiology (53). Hippocampal volume has repeatedly been implicated in the pathophysiology of MDD (54) and changes in structure and function of the hippocampus are associated with depressive symptoms across psychiatric diagnoses, e.g. in schizophrenia (55), and are even present in subclinical depression (56). Moreover, hippocampal volume is associated with antidepressant response to traditional antidepressants (57) and ketamine (58). Preclinical data suggest that ketamine induces synaptic and vascular alterations of the hippocampus 24 h after administration (59). However, the impact of reduced GABA+/tCr ratio 2 h after administration on ketamine-induced hippocampal neuroplasticity remains to be elucidated.

We did not observe a statistically significant association between plasma levels of major ketamine metabolites (norket, dhnk) and changes in neurotransmitter ratios. This is in line with a previous investigation by Milak et al. (11) that did not observe a correlation between norket and dhnk levels and Glx and GABA levels during administration. While antidepressant-like effects of ketamine metabolites were demonstrated in preclinical trials, the role of glutamate and GABA in their effects is currently not clear (60). Rodent studies show promising clinical antidepressant effects for hydroxynorketamine (hnk). However, we did not assess this metabolite in our study because levels typically peak 24 h after ketamine application (61).

Strengths of this investigation include use of a novel multivoxel MRSI sequence that allows for the hitherto regionally most extensive simultaneous assessment of human *in vivo* glutamatergic and GABAergic effects after ketamine exposure. Assessment of effects when clinical antidepressant properties emerge allows for probing of the correlates of antidepressant improvement itself while complementing existing literature on more short- and long-term changes. We thus contribute to a chronological framework for understanding ketamine’s glutamatergic and GABAergic effects.

Our study is not without limitations. This investigation does not follow a randomized, controlled study design. However, given ketamine’s unambiguous dissociative effects, an adequate control condition is challenging (62) and application of GABAergic compounds such as benzodiazepines, as performed in previous trials (2) is particularly questionable when GABA levels are assessed. The sample size (n = 25) of our investigation should be considered a limitation, nevertheless it exceeds those of the majority of previous pharmacological MRS studies (9–12, 15, 20, 21, 63) and is a result of our resource intensive methodology. In this context, the inclusion of imputed MRS data should also be discussed. We performed stringent quality control (CRLB thresholds) on MRS data and while this results in missing values, imputation allows for application of statistical procedures. However, it should be taken into consideration that mean imputation as performed reduces variance and may thus make subthreshold effects more pronounced. Extent of missing- and subsequently imputed values within an ROI may thus affect results. Moreover, the applied multivoxel MRSI sequence does not allow for discrimination of Glu and Gln, thus Gln/Glu is not assessed. Furthermore, signal spill-over from adjacent voxels cannot be excluded. Lastly, venous blood samples were not acquired during performance of MR measurements. This issue was, however, circumvented through interpolation of plasma curves.

In conclusion, we demonstrate a statistically significant decrease in GABA+/tCr in the hippocampus 2 h after ketamine infusion in healthy individuals. No statistically significant changes in Glx/tCr and GABA+/Glx were observed in any of the assessed ROIs. These results suggest changes to GABA turnover in the subacute time period after ketamine administration, in accordance with preclinical data (43, 44). Our findings, acquired during the time-period when antidepressant effects emerge, highlight a potential role for hippocampal GABAergic neurotransmission in ketamine’s antidepressant effects, though validation in MDD is required. This study thus contributes to our understanding of the chronology of neurotransmitter changes after ketamine administration.

## Data Availability Statement

The datasets presented in this article are not readily available due to ethical reasons. Please contact marie.spies@meduniwien.ac.at for questions.

## Ethics Statement

This study was reviewed and approved by the Ethics Committee of the Medical University of Vienna. The participants provided their written informed consent to participate in this study.

## Author Contributions

MS and RL designed the study. LS, BS, and MS wrote the manuscript. Data analyses were performed by BS under supervision of MS, MK, and WB and contribution of LS. LS, PH, PT, MEK, MW, and MS provided medical support. VR provided administrative support. BR and TS performed analyses of plasma levels of ketamine and its metabolites. BS performed MR measurements. MS was scientific supervisor of the study and principal investigator. All authors contributed to the article and approved the submitted version.

## Funding

This project was funded by Brain and Behavior Research Foundation (formerly NARSAD) Young Investigator grants to MS (23741) and PB (27238) as well as grants from the Austrian Science Fund (FWF, KLI516) and the Vienna Science and Technology Fund (WWTF, CS18-039) awarded to RL. Additional support was provided by the Medical University of Vienna’s Medical Imaging Cluster and a grant from the Austrian Science Fund (FWF, P30701) awarded to WB. LS and MK are recipients of DOC fellowships of the Austrian Academy of Sciences at the Department of Psychiatry and Psychotherapy, Medical University of Vienna. PB is recipient of a Marie Skłodowska-Curie Individual Fellowship (846793). MEK is a Medical University of Vienna MD PhD Excellence Program awardee. Funding sources had no further role in study design, collection, analyses and interpretation of data, writing of the report; nor in the decision to submit the paper for publication.

## Conflict of Interest

RL received travel grants and/or conference speaker honoraria within the last 3 years from Bruker BioSpin MR, Heel, and support from Siemens Healthcare regarding clinical research using PET/MR. He is shareholder of BM Health GmbH since 2019. MS has received speaker honoraria from Janssen and Austroplant as well as travel grants and/or workshop participation from Janssen, Austroplant, AOP Orphan Pharmaceuticals, and Eli Lilly.

The remaining authors declare that the research was conducted in the absence of any commercial or financial relationships that could be construed as a potential conflict of interest.
