# Supplementary material for: Effect of Ketamine on Limbic GABA and Glutamate: A Human In Vivo Multivoxel Magnetic Resonance Spectroscopy Study
Source: Front Psychiatry. 2020 Sep 8;11:549903. doi: 10.3389/fpsyt.2020.549903 (PMC7507577; doi:10.3389/fpsyt.2020.549903)
Supplement: Supplementary file 1 [file DataSheet_1.docx]

SUPPLEMENT

**Table S1:** Mask volume and mean number of MRS voxel per ROI

|  | volume (cm³) | interpolated voxel |
| --- | --- | --- |
| Thalamus | 17.6 ± 1.6 | 26031 ± 3823 |
| Hippocampus | 9.3 ± 0.9 | 14179 ± 1461 |
| Insula | 16.4 ± 1.7 | 19102 ± 3550 |
| Putamen | 10.7 ± 1.7 | 13932 ± 2454 |
| rACC | 5.5 ± 0.9 | 10891 ± 1649 |
| cACC | 4.1 ± 0.6 | 5886 ± 1509 |
| PCC | 7.2 ± 0.9 | 7128 ± 1504 |

Mean (± standard deviation) mask volume (cm³) and the number of interpolated voxels for each ROI used in the analysis. rACC = rostral anterior cingulate cortex, cACC = caudal anterior cingulate cortex, PCC = posterior cingulate cortex.

**Table S2: Mean plasma levels of ketamine, norketamine and dehydronorketamine.**

Time indicates minutes after start of ketamine infusion.

|  | ket (ng/ml) | norket (ng/ml) | dhnk (ng/ml) |
| --- | --- | --- | --- |
| Time point | (mean ± SD) | (mean ± SD) | (mean ± SD) |
|  | | | |
| 20' | 152.77 ± 45.41 | 16.94 ± 6.98 | 1.22 ± 1.27 |
| 40' | 231.14 ± 63.80 | 52.15 ± 18.16 | 5.80 ± 5.08 |
| 50' | 254.60 ± 69.00 | 73.59 ± 22.11 | 8.87 ± 7.37 |
| 55' | 229.63 ± 67.44 | 85.62 ± 26.69 | 9.93 ± 7.47 |
| 60' | 213.37 ± 55.67 | 99.88 ± 32.03 | 12.48 ± 9.39 |
| 70' | 193.15 ± 69.72 | 107.23 ± 36.03 | 13.82 ± 9.85 |
| 80' | 169.97 ± 71.30 | 105.76 ± 32.13 | 14.56 ± 10.18 |
| pre-MR | 119.84 ± 23.67 | 111.19 ± 25.36 | 16.49 ± 10.03 |
| post-MR | 68.23 ± 14.38 | 95.22 ± 25.21 | 15.54 ± 8.59 |

Ket = ketamine, norket = norketamine, dhnk = dehydronorketamine.


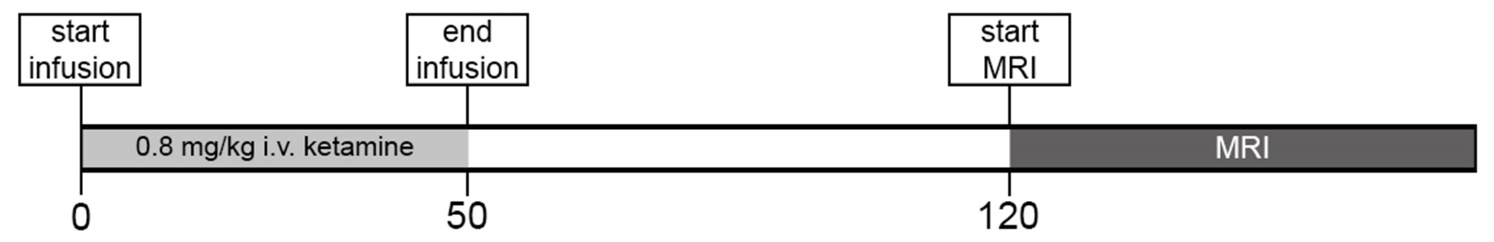


Figure S1: Schematic illustration of MRI2 protocol. Subjects received 0.8mg/kg bodyweight racemic ketamine intravenously over a period of 50 minutes. MRI acquisition started approximately 120 minutes and MRSI data acquisition started 137.44 ± 3.43 (mean ± SD) minutes after initiation of the ketamine infusion.


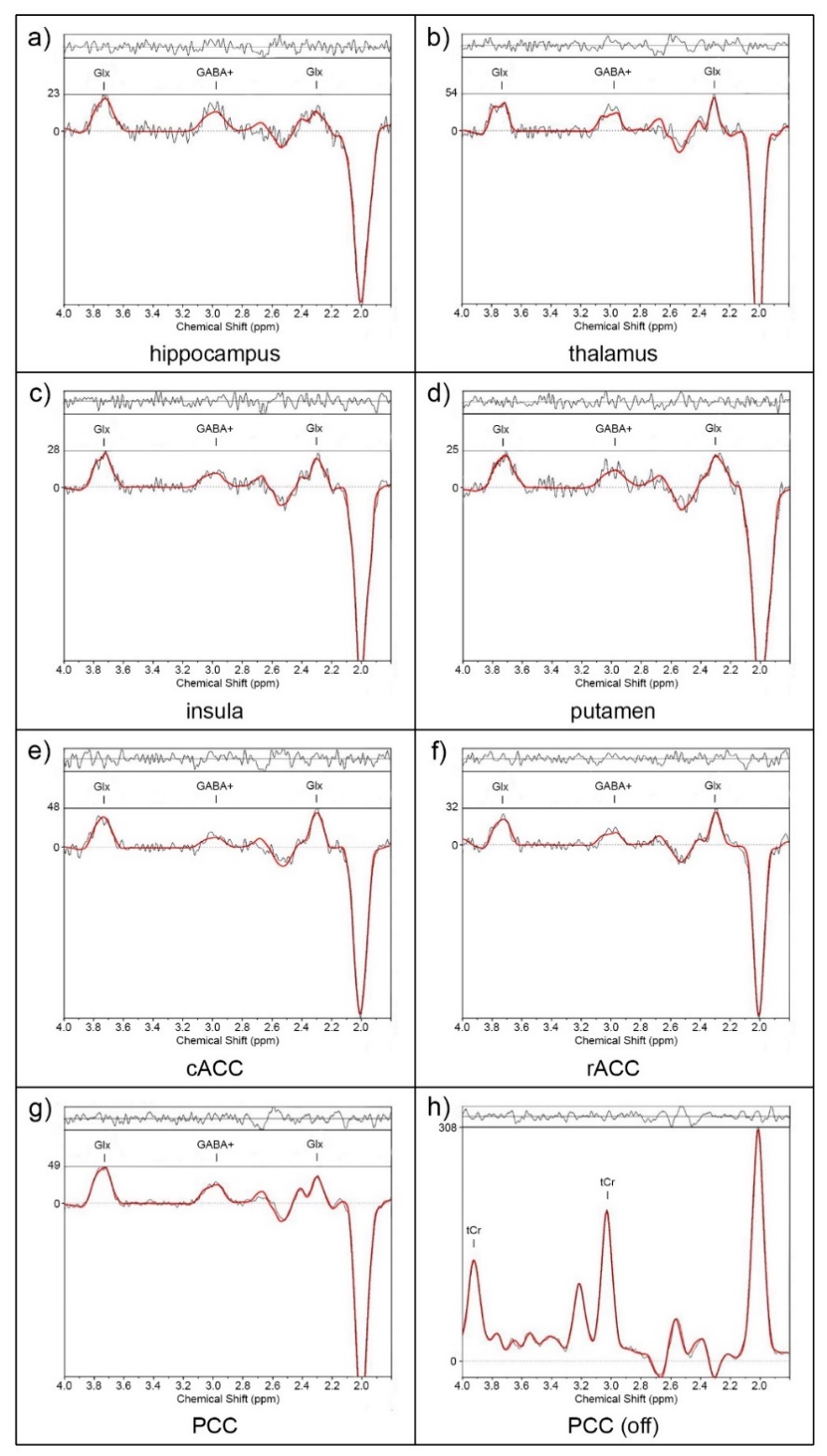


**Figure S2:** **Exemplary *in vivo* proton MR spectra obtained from all regions with the GABA‐editing MEGA‐LASER 3D MRSI sequence**. The LCModel fit of metabolites in the difference (difference spectrum; subtraction of EDIT‐ON and EDIT‐OFF) spectrum is shown (a-g) in addition to the respective EDIT-OFF spectra of the PCC (h). MRSI = multi-voxel 3D-magentic resonance spectroscopy imaging. rACC = rostral anterior cingulate cortex, cACC = caudal anterior cingulate cortex, PCC = posterior cingulate cortex.
